# Post‐Coronavirus disease syndrome (post‐COVID‐19) syndrome: A case report

**DOI:** 10.1002/ccr3.6719

**Published:** 2022-12-20

**Authors:** Rukshar Thapa, Prashant Pant, Pragyat Singh, Sagun Karki, Kriti Thapa, Saman Aryal, Oshan Shrestha

**Affiliations:** ^1^ Medical Officer, Clinic Health Care Center Kathmandu Nepal; ^2^ Department of Anesthesia and Critical Care Unit Karnali Academy of Health Sciences Jumla Nepal; ^3^ Medical Officer, Green City Hospital Kathmandu Nepal; ^4^ College of Medicine Nepalese Army Institute of Health Sciences Kathmandu Nepal; ^5^ Shree Birendra Hospital Nepalese Army Institute of Health Sciences Kathmandu Nepal; ^6^ Medical Researcher, Road Safety Research Nepal Automobile Association Kathmandu Nepal

**Keywords:** coronavirus, COVID‐19, post‐COVID, syndrome

## Abstract

Post‐COVID syndrome, a cluster of symptoms that develops or persists even after the recovery from COVID‐19 or viral clearance, can have multi‐system manifestations. This entity should be considered in patients who recently tested positive for COVID‐19 after ruling out other possible obvious causes. Its management should involve a multidisciplinary approach.

## Graphical abstract

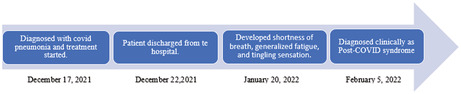

## 1 INTRODUCTION

The pain inflicted by the COVID‐19 pandemic on the world is still fresh. It is to be acknowledged that many people lost their lives to this disease but the vast majority of patients had mild symptoms.^1^ Among those who recovered and survived the wave of COVID‐19, irrespective of the severity of the disease, were seen to develop new symptoms after the recovery. These symptoms persist even after viral clearance and have no other explanation.^2^ This was dismissed by scientific and medical communities at first, but later this got established as post‐COVID syndrome or long COVID.^3^, ^4^ The most common symptoms of the syndrome include fatigue and dyspnoea, while, symptoms of neuropsychiatric disorders, neurological disorders, insomnia, autonomic dysfunction, smell and taste dysfunctions, cough, hair loss, myalgia, wheezing, and cardiac and gastrointestinal issues are also reported in lesser frequencies.^2^, ^3^

In this case report, we present such a case of post‐COVID syndrome in which the patient developed shortness of breath, fatigue, myalgia, and tingling sensation in hands and feet after recovery and these persisted after viral clearance. This case report is in line with CARE guidelines.^5^

## 2 CASE REPORT

A 34‐year‐old female, with no history of medical/surgical/neuropsychiatric disorders and no family history of chronic illnesses, initially presented with low‐grade intermittent fever (T_max_ of 100.5 F) and non‐productive cough which had acute onset. She also showed concern for her few episodes of vomiting (non‐bilious and non‐projectile), chest pain, and shortness of breath that she had developed. All of her symptoms started 7 days before her presentation to the hospital except for vomiting, which started 2 days before the presentation. The patient was then diagnosed with COVID‐19 pneumonia which was confirmed by laboratory workup and imaging. Treatment was started for her condition and the patient was consequently discharged after proper management and improvement of her symptoms. However, approximately 1 month after the discharge she began developing symptoms of shortness of breath, generalized fatigue, and tingling sensation in her hands and feet bilaterally. She also had complaints of disturbed sleep and feeling of apprehension.

### 2.1 Timeline

**FIGURE 1:**
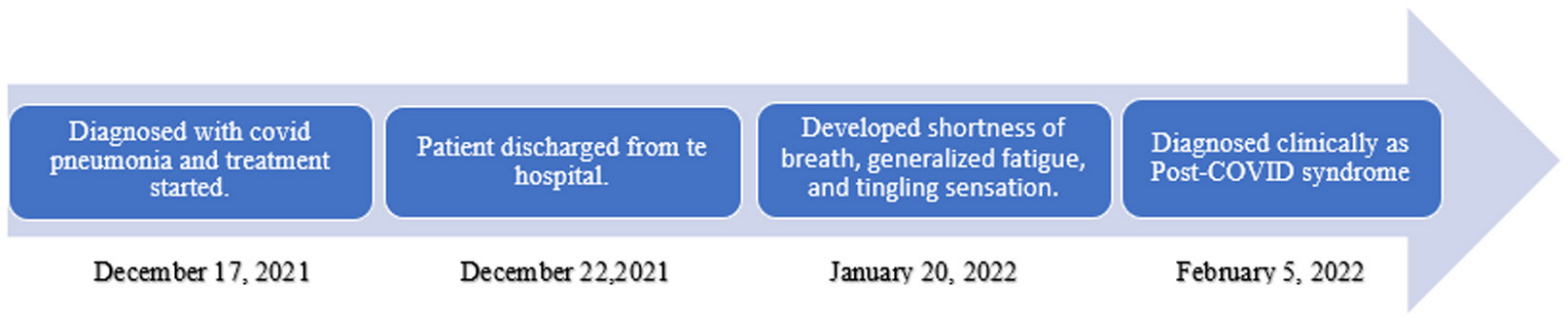
Timeline showing major events

All the major clinical events from the diagnosis of COVID‐19 to its recovery and development of new symptoms after the recovery are shown in a timeline in Figure 1 **.**

### 2.2 Diagnostic assessments

**FIGURE 2:**
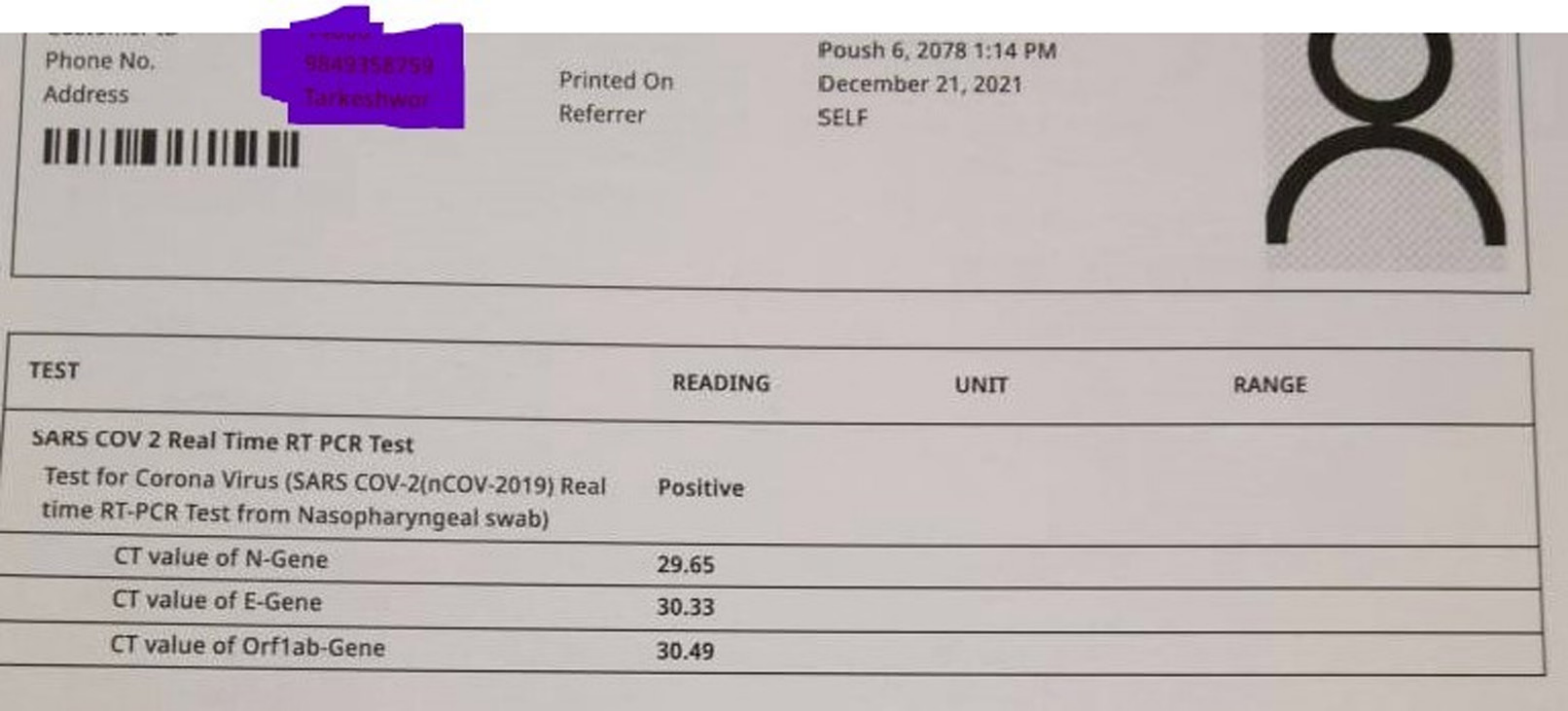
Initial RT PCR report

**FIGURE 3:**
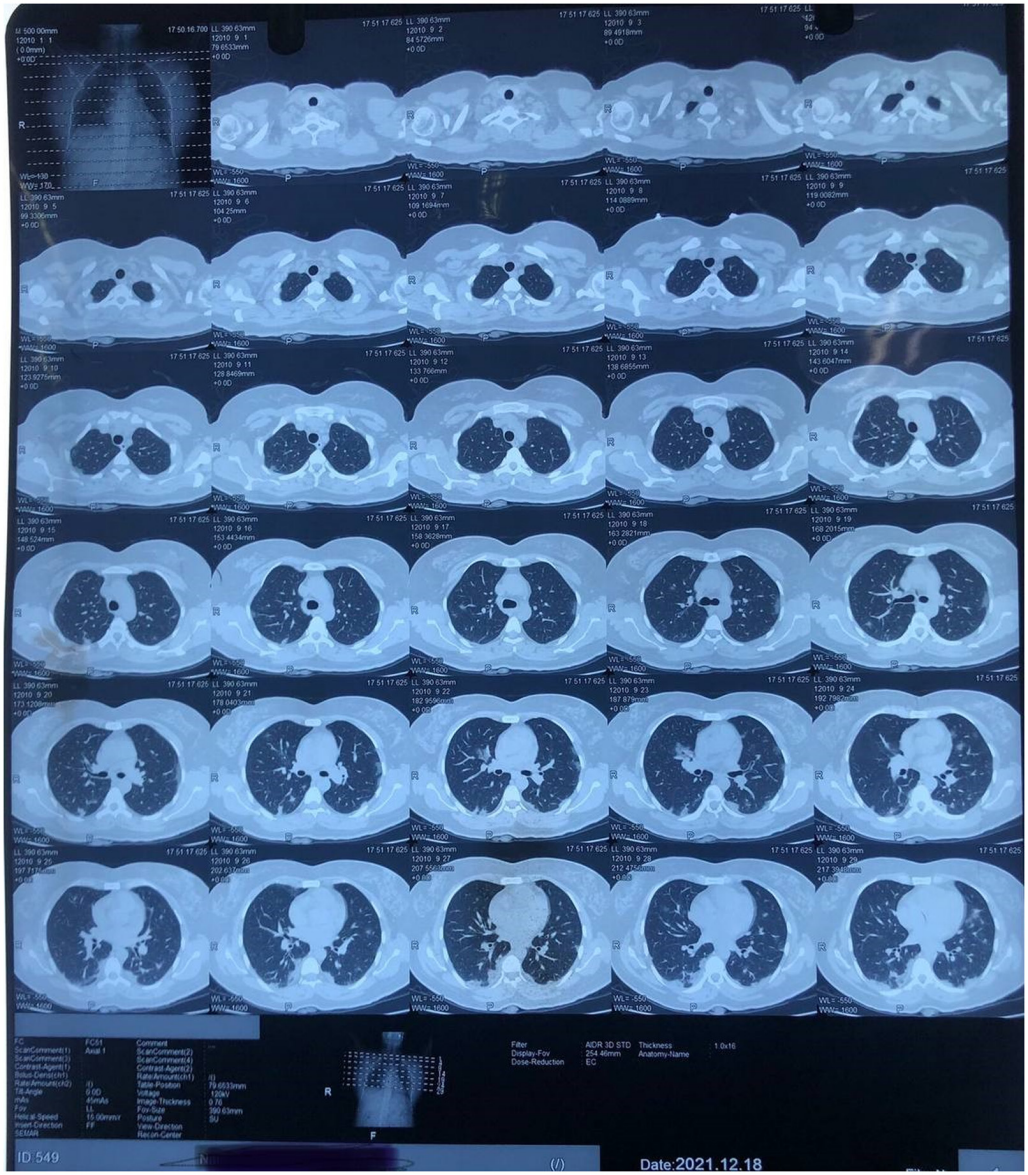
HRCT showing pleural effusion

**FIGURE 4:**
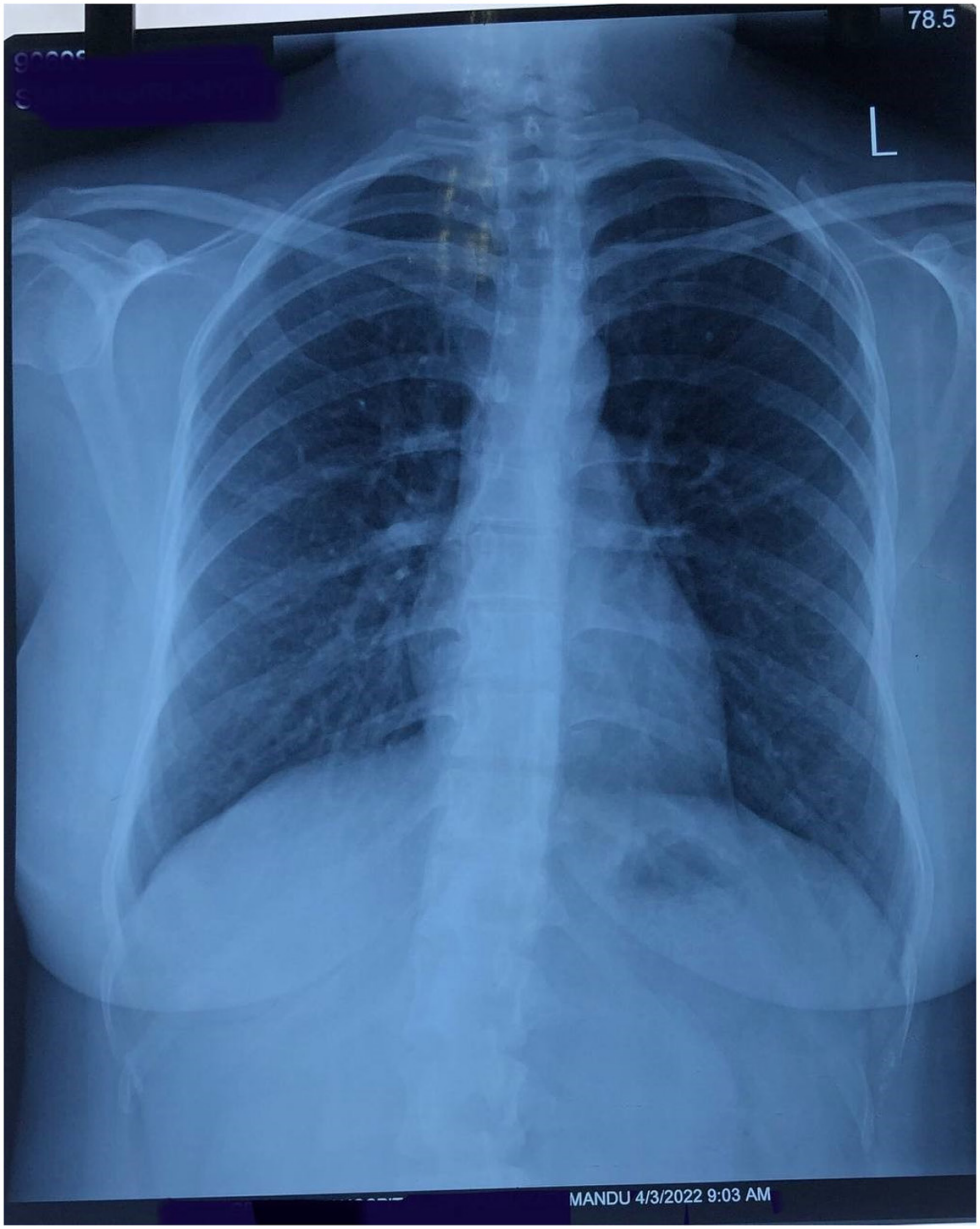
Chest X‐ray showing no sign of pleural effusion

**FIGURE 5:**
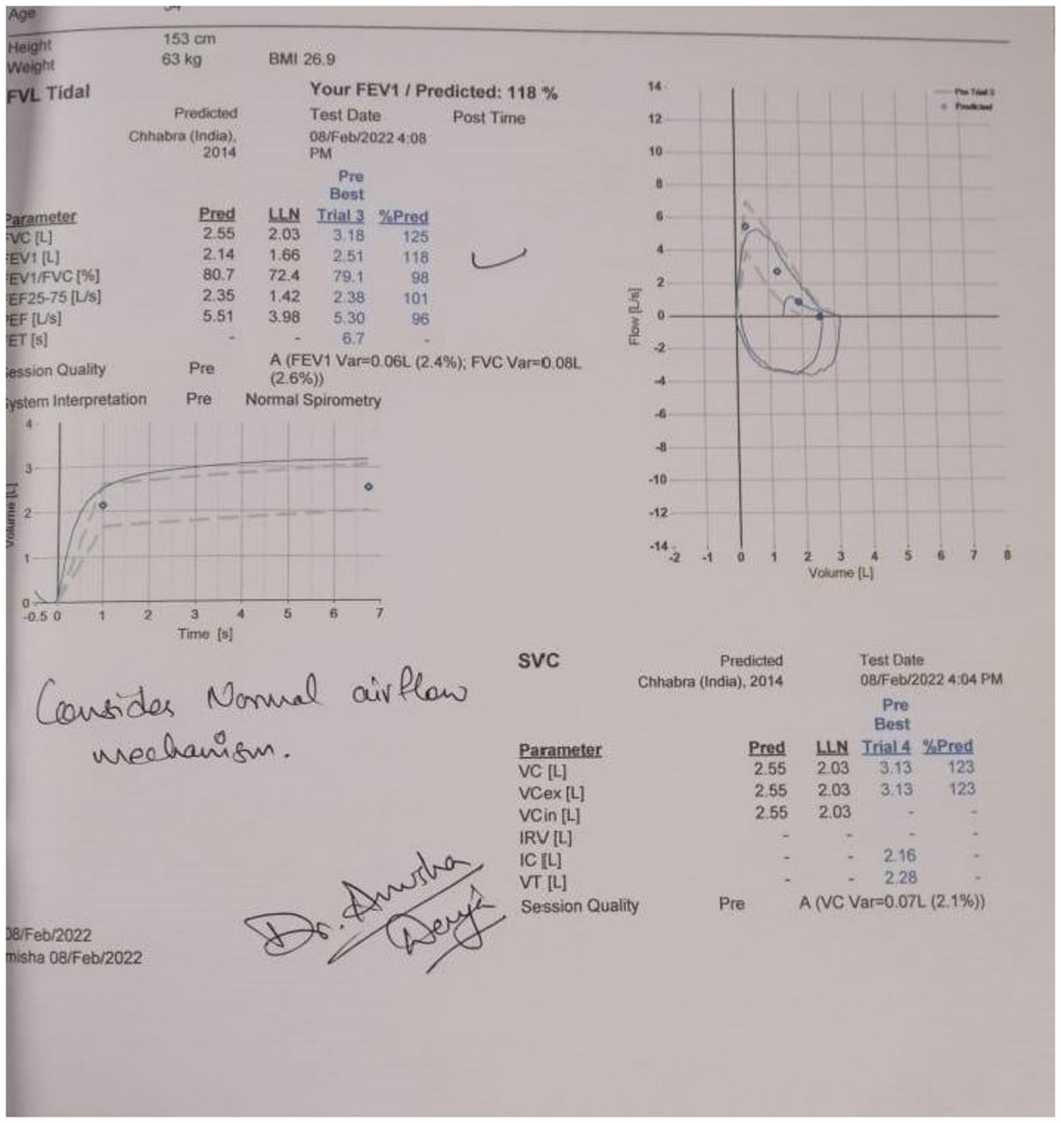
Spirometry results

**FIGURE 6:**
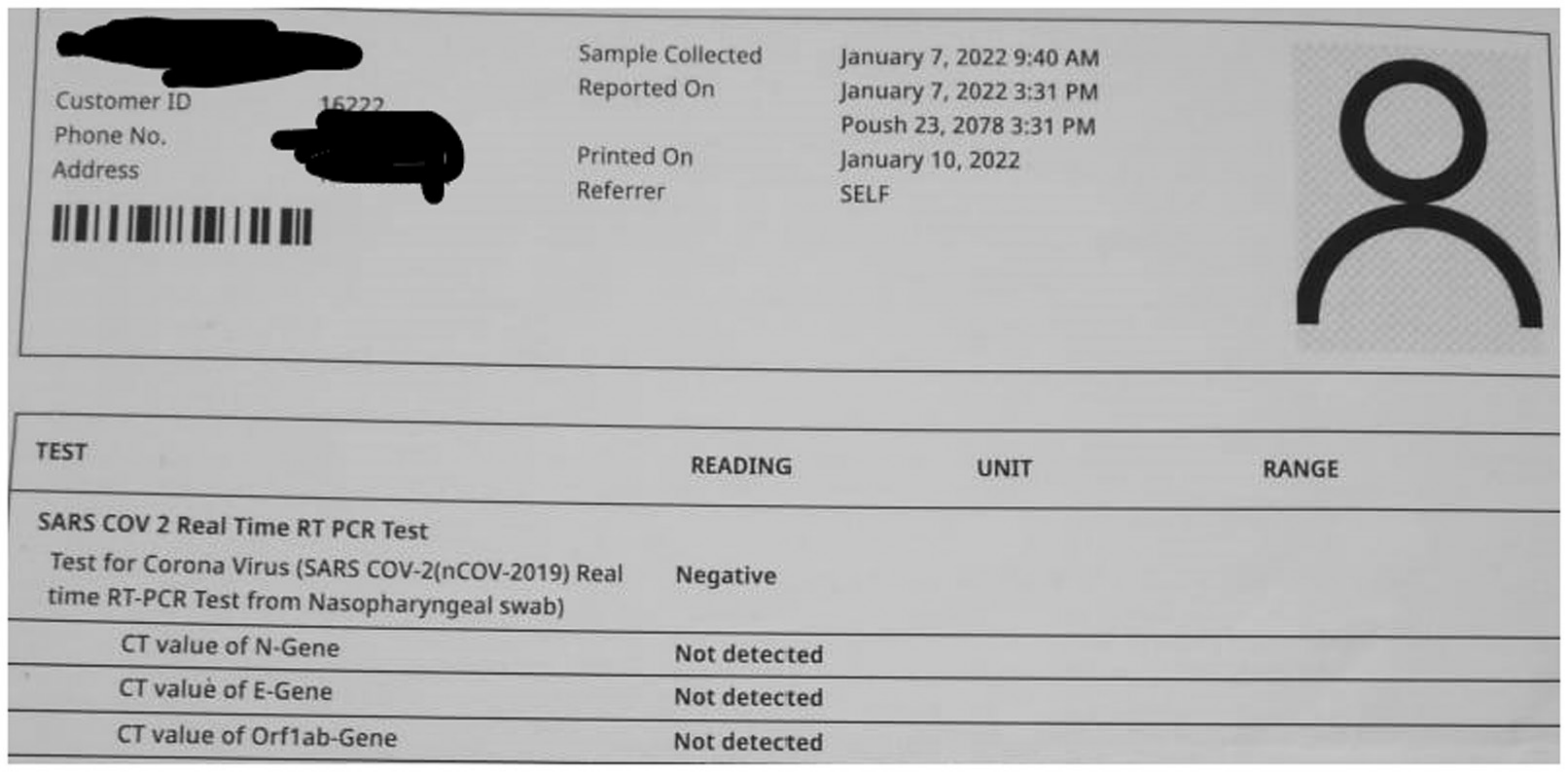
RT PCR showing a negative result

On clinical examination, all the findings were normal except for bilaterally decreased breath sounds. The patient was first diagnosed with COVID Pneumonia after a confirmatory polymerase chain reaction (PCR) and high‐resolution computed tomography (HRCT) of the chest. The report of RT‐PCR is shown in Figure 2 and HRCT is shown in Figure 3. Later, when the patient developed symptoms after a month then laboratory and imaging workups were carried out. Chest x‐ray, electrocardiogram, spirometry, thyroid function test, ultrasonography of the abdomen and pelvis, echocardiography, and vitamin B12 levels showed no abnormalities (Figure 4, Figure 5, and Figure 6).

After considering the history of COVID‐19, ruling out all the possible causes, imaging, and laboratory workups, the diagnosis of post‐COVID syndrome (with the predominance of neuropsychiatric symptoms) was made clinically.

### 2.3 Treatment

Pharmacological treatment of the case included flexion tablets (combination of 325 mg of paracetamol and 400 mg of ibuprofen), escitalopram tablets of 10 mg, clonazepam tablets of 0.25 mg, pantoprazole tablets of 40 mg, and cyanocobalamin tablets of 1 mg. The duration of treatment was for 10 days. Besides pharmacological treatment, the patient was also taught breathing exercises and was recommended for 5–10 min a day. Treatment was started after multidisciplinary consultation.

### 2.4 Follow‐up

The patient party was counseled on the course and prognosis of her condition and was advised to seek multimodal‐multispecialty care as needed. The patient is seeing improvement in her symptoms and is advised to report after 10 days and earlier if she develops new symptoms are the existing symptoms worsen.

## 3 DISCUSSION

This article has presented a case of a 34‐year‐old female who developed symptoms (shortness of breath, generalized fatigue, tingling sensation, disturbed sleep, and feeling of apprehension) after the recovery from COVID‐19. All the possible causes were ruled out and the development of such symptoms even after viral clearance (after COVID‐19 recovery), led to the conclusion of post‐COVID syndrome. The entity itself, post‐COVID syndrome, is a relatively new topic and its description is not uniform among various studies due to which there is no absolute definition of post‐COVID syndrome.^6^ However, guidelines from National Institute for Health and Care Excellence (NICE) state that post‐COVID syndrome develops after an infection consistent with COVID‐19 and continue for more than 12 weeks, and is not explained by alternative diagnosis. The guideline also states that post‐COVID syndrome can be considered before 12 weeks after assessing and ruling out other possibilities.^7^

Post‐COVID syndrome includes symptoms like fever, sore throat, fatigue, and neurological and neuropsychiatric symptoms.^2^, ^3^ Some authors have also put forward a question of these symptoms occurring as a result of post‐traumatic distress and having a neuropsychiatric predominance.^8^ Our patient also developed sleep disturbances and anxiety and the absence of abnormalities in other examinations and laboratory workups have supported this question. One study followed‐up survivors of severe acute respiratory syndrome (SARS) infection for 4 years and found that chronic fatigue and psychiatric conditions remained significant.^9^ So, management of mental health morbidities with rehabilitation remains an important aspect of the treatment of post‐COVID syndrome. Another question is if the symptoms persist or arise again after recovery. In this case, the patient developed symptoms approximately 1 month after the recovery from COVID‐19. Symptoms can occur in absence of no abnormalities in clinical examination, laboratory workups, and radiology, like that in this case. In such instances, the patient should be taken seriously. One study has reported an experience of a patient who was told off by the health care providers by stating that it is all in her head only.^10^ It is to be taken into account that post‐COVID syndrome can have neuropsychiatric predominance and support through a multidisciplinary approach is crucial, including emotional support. Regarding the risk factors, various studies have put forward possible risks for the development of post‐COVID syndrome. Out of those risk factors, female sex has been reported as a common factor in various studies.^3^, ^11^, ^12^ Previous history of mental illness and comorbidities are also mentioned as risk factors but our female patient had no such history. With not much research on the topic and variability of symptoms in post‐COVID syndrome, there is no standardized treatment guideline. In this case, patient care was focused on relieving the patient's symptoms, improving her quality of life through improving her sleep, and rehabilitation through breathing exercises.

This case report can act as a means of communicating our findings to share our experiences so that the medical community is informed. Evidence gaps remain in the development, risk factors, and treatment of the condition. This article aims to motivate the medical community to do further research on the topic to establish more concrete evidence in the age of evidence‐based practice.

## 4 CONCLUSION

Post‐COVID syndrome should be considered in patients recently infected with COVID‐19 and presenting with shortness of breath, generalized fatigue, sleep disturbances, autonomic symptoms, and neuropsychiatric symptoms. Symptoms can have neuropsychiatric predominance, so the management should be directed accordingly with multidisciplinary consultation.

## AUTHOR CONTRIBUTIONS

Rukshar Thapa (RT), Prashant Pant (PP), Pragyat Singh (PS), and Oshan Shrestha (OS) contributed to the conception and design of the study. RT, PP, PS, and Kriti Thapa (KT) contributed to acquiring patients' detailed information. Sagun Karki (SK), Saman Aryal (SA), and OS performed the literature review and OS contributed to the initial manuscript drafting. All authors were involved in revising the manuscript and approving the final version.

## CONFLICT OF INTEREST

No conflict of interests.

## ETHICAL APPROVAL

N/A

## CONSENT

Written informed consent was obtained from the patient for the publication of this case report and accompanying images. A copy of the written consent is available for review by the Editor‐in‐Chief of this journal on request.

## CLINICAL TRIAL REGISTRATION

N/A

## PATIENT PERSPECTIVE

The patient is distressed due to her condition, especially due to disturbed sleep and fatigue. Proper counseling and improvement of symptoms after initiating treatment have brought a positive outlook in the patient.

## Data Availability

All the findings are present within the manuscript.
